# Response to case report: Airway autoimmune responses in severe eosinophilic asthma following low-dose Mepolizumab therapy

**DOI:** 10.1186/s13223-017-0217-6

**Published:** 2017-11-03

**Authors:** I. J. Pouliquen, P. Howarth, D. Austin, G. Gunn, E. Meyer, R. G. Price, E. Bradford

**Affiliations:** 1 Clinical Pharmacology Modelling & Simulation, GlaxoSmithKline, Uxbridge, UK; 2 Respiratory Franchise, GlaxoSmithKline, Brentford, UK; 3 Immunogenicity and Clinical Immunology, GlaxoSmithKline, King of Prussia, PA USA; 4 Clinical Statistics, GlaxoSmithKline, Uxbridge, UK; 5 Respiratory Therapeutic Area, GlaxoSmithKline, Research Triangle Park, NC USA

**Keywords:** Case report, Severe eosinophilic asthma, Mepolizumab, OCS, IL-5

To the Editor,

We read with interest the case report and accompanying discussion published by Mukherjee et al. (AACI 2017;13:2) of a 62-year old woman diagnosed with severe eosinophilic asthma. This clinical case presents a patient with progressive deterioration in FEV_1_ function since 2011 with no improvement observed while receiving OCS, hydroxyurea or imatinib therapy. On this background of deterioration, the patient entered the double-blind placebo controlled clinical trial MEA115575 and received mepolizumab 100 mg s.c. every 4 weeks. Further deterioration in FEV_1_ coincided with the (protocol defined) reduction in prednisolone during the study, and neither intravenous solumedrol nor pre-study prednisolone doses improved FEV_1_ to pre-study values. During the open-label mepolizumab extension study MEA115661 the patient’s clinical status was unchanged. In January 2015, 9 months after their last dose of mepolizumab, the patient further deteriorated whilst receiving azathioprine immunosuppressive therapy. This clinical case presentation clearly underlines the aggressive nature of this patient’s asthma and their inadequate response to therapy.

Mukherjee et al. suggest that the dose of mepolizumab, and subsequent concentration in the airways, was too low to neutralize completely the high levels of IL-5 in the airways of the patient. They further advance the hypothesis that equimolar concentrations of mepolizumab and IL-5 in the lung may have led to the formation of immune complexes contributing to uncontrolled airway eosinophilic inflammation.

Since the concentration of mepolizumab has never been measured in the lung, it is not possible to refute, or support this hypothesis. The tissue partitioning of IgG1 antibodies is, however, better-understood and would suggest that equimolarity is extremely unlikely based on known IL-5 levels. Furthermore, the structure of mepolizumab-IL-5 complexes are concentration-dependent; with one mepolizumab molecule binding to two IL-5 at low drug concentrations, and two mepolizumab molecules binding to two IL-5 in its most stable form when drug is in only three-fold excess. Neither of these simple complexes would promote the formation and deposition of large immune complexes in tissue, as suggested by Mukherjee et al.

This patient’s initial response to mepolizumab therapy, as attested by profound reductions in both blood and sputum eosinophils, with subsequent loss of responsiveness (despite ongoing mepolizumab therapy and absence of neutralising antibodies), raises an important question. Is there a sub-group of patients with particular features and notably those who require high dose oral steroids, generally of 20 mg or more daily to control their disease, that differentiate them from the rest of the severe asthma patients? Intake of high dose oral steroids could obscure the true underlying and hypereosinophilic nature of the disease, which is revealed following oral steroids tapering in those patients. This group of patients could potentially represent a form of severe asthma associated with an eosinophilic granulomatosis with polyangiitis (EGPA) syndrome restricted to the lung. EGPA patients were shown to respond to a mepolizumab dose of 300 mg s.c. every 4 weeks [1], however no dose ranging investigation was carried out. In such a specific severe asthma phenotype it cannot, therefore, be ruled out that the mepolizumab dose of 100 mg s.c. every 4 weeks recommended in severe asthma might be too low, preventing successful reduction of oral steroids due to concurrent localized EGPA. It is also possible that some patients cannot be fully controlled with therapies reducing eosinophils. Regardless, this clinical case opens the hypothesis of a potential distinct group of severe asthma patients of utmost interest and warrants the need for further clinical investigation.

## References

[1] Wechsler ME, Akuthota P, Jayne D, Khoury P, Klion A, Langford CA, Merkel PA, Moosig F, Specks U, Cid MC, Luqmani R, Brown J, Mallett S, Philipson R, Yancey SW, Steinfeld J, Weller PF, Gleich GJ (2017). Mepolizumab or placebo for eosinophilic granulomatosis with polyangiitis. N Engl J Med.

